# Influence of Solid Alkaline Photocatalysts Irradiated with UV Light on Fuel Properties of Palm Oil Biodiesel

**DOI:** 10.1021/acsomega.4c04991

**Published:** 2024-09-09

**Authors:** Cherng-Yuan Lin, Shun-Lien Tseng

**Affiliations:** Department of Marine Engineering, National Taiwan Ocean University, Keelung 20224, Taiwan

## Abstract

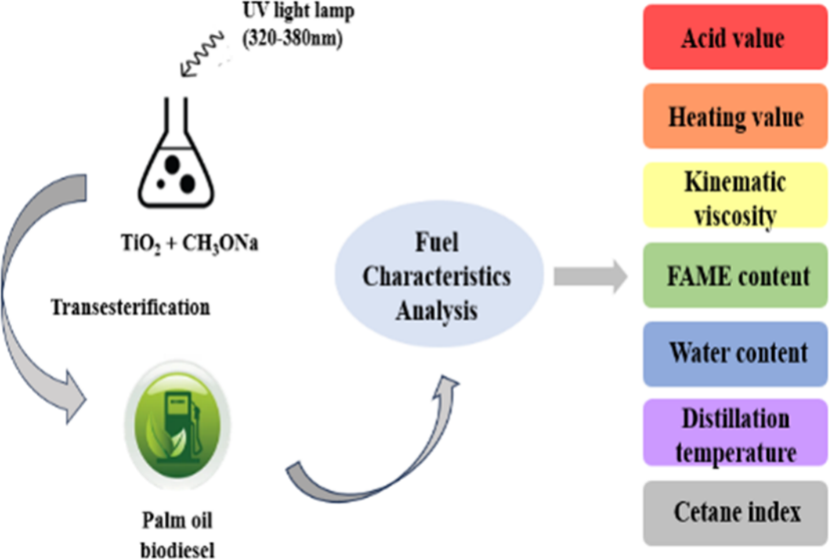

TiO_2_ nanoparticles are full of porosity that can be impregnated with a strong alkaline catalyst CH_3_ONa to form a TiO_2_/CH_3_ONa catalyst. TiO_2_ of the anatase phase, which is a semiconductor material, has been a prominent photocatalyst due to its excited photocatalyst activity, chemical and biological stability, and nontoxicity. The CH_3_ONa compound has been widely used as a catalyst for transesterification. Although the synthesized photocatalyst TiO_2_ powder with CH_3_ONa is anticipated to greatly enhance the transesterification efficiency, leading to improving biodiesel properties, relevant studies have not been found. After the photocatalyst was prepared, a reactant mixture of palm oil, methanol, and heterogeneous catalyst TiO_2_/CH_3_ONa was illuminated by ultraviolet (UV) light from light-emitting diode (LED) lamps. The experimental results revealed that the formation of fatty acid methyl esters was significantly increased to 98.4% with ultraviolet-light illumination for the molar ratio of methanol/palm oil equal to 6 and 3 wt % catalyst addition. The decrease of the catalyst amount to 2 wt % resulted in a slight decrease of the fatty acid methyl esters to 97.06 wt %. The lowest kinematic viscosity and acid value and the highest distillation temperature, heating value, and cetane index were observed under the above reaction conditions. The distillation temperature and cetane index were increased while the acid value was decreased under ultraviolet illumination on the reactant mixture. Consequently, the optimum preparing conditions for biodiesel production were 6 and 3 wt % for the molar ratio of methanol/palm oil and catalyst addition under UV-light irradiation.

## 1 Introduction

The use of biodiesel as an alternative fuel to diesel fuel in combustion engines and boilers has been fast developing. The conversion from low-cost degraded feedstock oils to high-quality biodiesel might rely on the addition of adequate and effective catalysts into the reactant mixture.^1^ Strong alkaline, acid, or enzyme catalysts could be used for the catalyst to enhance the transesterification reaction; however, only strong alkaline catalysts, particularly sodium methoxide (CH_3_ONa), potassium hydroxide (KOH), sodium hydroxide (NaOH), etc., are adaptable to mass-sized and fast rate industrial biodiesel production processes.^2^ Since alcohols are not well-miscible with animal fats or vegetable oils during transesterification for biodiesel production, this leads to a decrease in the reaction efficiency.^3−5^ The addition of some adequate cosolvents such as tetrahydrofuran (THF), diethyl ether, methyl tertiary-butyl ether (MTBE), etc. to the reactant mixture of lipids and alcohols can improve their solubility extent. Some auxiliary technologies including the supercritical methanol (SCM) process, microwave irradiation, ultrasound vibration, etc. can be used instead of conventional stirring reactors to increase the contact area among the reactant mixtures, resulting in an enhancement of the reaction efficiency.

In comparison with the use of a homogeneous catalyst, a heterogeneous solid-state catalyst requires more careful control for operating parameters such as the reaction temperature, pressure, and particle size and generally has a lower conversion ratio from feedstock oils through the transesterification reaction. However, a heterogeneous catalyst is more readily removed from the crude product mixture after completing the transesterification reaction and to enhance the final product purity.^6^ The removed catalyst can be recycled for the next production process so that the consumed catalyst can be reduced, curtailing the widely used industrial biodiesel production.

Two different types of catalysts could be synthesized to prepare a heterogeneous catalyst to combine various catalyst functions. One catalyst could be sintered with another one to have synergistic catalyst effects so that the transesterification rate for biodiesel production can be further accelerated in comparison with a single catalyst.^7^ Some crystalline metal oxides such as TiO_2_, Fe_3_O_4_, SiO_2_, and CeO can be used as carriers to embed strong acid or alkaline catalysts, which are then calcined to be synthesized catalysts. In particular, TiO_2_ has semiconductor material characteristics and consists of many pores and holes in its nanometered structure. The porosity of TiO_2_ provides an admissible space to fill in synthesized catalysts and to calcine them together. López et al.^8^ synthesized a sodium titanate catalyst via the sol–gel hydrothermal method. After titanium dioxide (TiO_2_) was synthesized with sodium hydroxide (NaOH), the TiO_2_/NaOH catalyst was added to the reactant to catalyze the transesterification reaction for biodiesel production. The highest conversion rate from soybean oil to biodiesel reached the highest 80% when the alcohol/oil molar ratio of 6, 55 °C of reaction temperature for 5 h, and a surface area of 217 m^2^/g of the TiO_2_/NaOH catalyst were used. Rahim et al.^9^ used a catalyst of TiO_2_ impregnated with calcium oxide (CaO) to catalyze the transesterification reaction for producing biodiesel. TiO_2_ generally played the role of a carrier to store the CaO catalyst. The conversion rate from waste cooking oil was significantly increased from 94.58% when only the calcium oxide (CaO) catalyst was used to 98.78% when the CaO catalyst was replaced with the synthesized catalyst TiO_2_/CaO. Hence, a synthesized catalyst of two adequate catalysts could effectively enhance the conversion rate of biodiesel in comparison with that of a single catalyst material. The optimum reaction time, molar ratio, and catalyst loading for the optimum conversion rate were 150 min, 15, and 2.3 wt %, respectively, for the TiO_2_/CaO catalyst. Zhang et al.^10^ also found that the combined catalysts like nanocatalysts Li/ZnO–Fe_3_O_4_, SO_4_/Mg–Al–Fe_3_O_4_, and nanocrystalline CaO, Sr–Al double oxides, and nanosulfated zirconia all can enhance the biodiesel yield above 98%.

TiO_2_ is a semiconductor material with an energy band gap of 3.2 eV. The photocatalytic reaction induced by TiO_2_ mainly comes from the transfer of electrons between the valence energy band and the conduction energy band.^11^ When TiO_2_ is irradiated by light carried with energy larger than the energy band gap of TiO_2_, the electrons in the valence energy band will jump into the conduction energy band, leading to the formation of empty holes, which are not filled with electrons in the valence energy band. The empty hole left is called an electron hole, which has the same mass as the electron but has opposite polarity.^12^ The main function of an electric hole is to carry charged particles in the semiconductors.^13^

The origin of the photocatalytic effect arises from the generation of charge carriers. Sahu et al.^14^ investigated the use of the photocatalysts TiO_2_/SO_4_ and ZrO_2_/SO_4_ to assist the transesterification of cotton seed oil containing a high free fatty acid content to produce biodiesel. Their experimental results showed that the reactant surface area covered by SO_4_ was directly proportional to the activity of the catalysts. A 90% high conversion ratio from the feedstock oil could be achieved by the catalytic effect of TiO_2_/SO_4_ at a specific surface area of 99.5 m^2^/g of SO_4_ coverage.

Sodium methoxide (CH_3_ONa) is considered a competitive, strong alkaline catalyst for transesterification. The synthesized CH_3_ONa filling in the pores of a promising crystalline carrier could promote the conversion rate from a feedstock oil to its biodiesel product. The photocatalyst TiO_2_/CH_3_ONa could be a promising catalyst combination to improve the feedstock oil conversion rate. The catalytic characteristics and auxiliary reacting machines are significant factors in determining not only the conversion rate from feedstock oil to the biodiesel product^15,16^ but also improving the fuel properties of the FAME formed from the transesterification reaction.

The catalyst [Fe_2_(SO_4_)_3_] was used to catalyze microwave irradiation-assisted transesterification. They found that the highest yield that achieved 98.66 wt % after the catalyst dosage of 2.96 wt % was used.^17^ Ionic liquid choline hydroxide (CHOH) was applied as a catalyst for the transesterification reaction of feedstock sunflower oil with methanol by Lima et al.^18^ They found that the highest ester yield of 95.05 wt % was obtained when 2 wt % catalyst dosage was added to the reactant mixture. The dosage amount of the catalyst for transesterification in the range between 1 and 3 wt % is found to cause a higher ester yield.^19^ Hence, the catalyst dosage was set between 1 and 3 wt % of the weight of feedstock oil in this study.

Heterogeneous catalysts bear specific features of surface and pore tuning characteristics, leading to the flexible selection of feedstock types for biodiesel manufacturing. High catalytic efficiency, substantial reusability, and sufficient durability of available heterogeneous catalysts are critical factors to improve biodiesel economic competence in alternative fuel markets.^20^ Hence, heterogeneous catalysts are considered promising solid catalysts for biodiesel synthesis.^21^ Noncatalytic supercritical methanol transesterification has been developed to manufacture biodiesel. The fuel properties of biodiesel through this process are almost not influenced by the existence of water, free fatty acids (FFAs), and feedstock compositions. It may only take a few minutes to complete the reaction,^22^ in comparison with 1 h by other traditional transesterification processes undertaken under atmospheric pressure. The major disadvantages of the supercritical transesterification processes lie in high apparatus construction and operating costs to maintain higher pressure and temperature of the equipment than the corresponding critical pressure and temperature of methanol, which are 81 bar and 513 K, respectively.^23^

Various combinations of calcined catalysts were observed to have high biodiesel yields for transesterifying different feedstocks. Pavlović et al.^24^ used a CaO/zeolite base catalyst to reach a biodiesel yield of 97.8%. The biodiesel yield from *Mespilus germanica* kernel oil through the transesterification catalyzed by a Al_2_O_3_/CaO nanocatalyst approached 96.68%.^25^ The application of a MgO/K_2_CO_2_ catalyst to the transesterification process of *Moringa olifiera* oil even achieved 99% biodiesel yield.^26^

Since sodium methoxide is the most widely used and promising strong alkaline catalyst for manufacturing biodiesel through transesterification processes,^27^ the titanium dioxide powder bears promising photocatalytic effects that can enhance the catalytic transesterification of CH_3_ONa.^28^ The adequate calcination of these two catalysts might significantly improve the biodiesel yield under the irradiation of ultraviolet light on the impregnated TiO_2_/CH_3_ONa catalyst. However, no study on the catalyst characteristics of the synthesized photocatalyst catalyst of TiO_2_/CH_3_ONa was reported and no biodiesel properties made from the photocatalyst TiO_2_/CH_3_ONa after being irradiated with ultraviolet (UV) light were analyzed in the literature.^29^ Due to the significance of the catalyst of TiO_2_ impregnated with CH_3_ONa on the biofuel product, the calcined catalyst of TiO_2_ with CH_3_ONa on UV-light irradiation was prepared to analyze the catalytic characteristics and fuel properties of biodiesel in this study. A solid strong alkali photocatalyst TiO_2_/CH_3_ONa was synthesized from TiO_2_ powder with a mean particle size of 22 nm sintered with sodium methoxide (CH_3_ONa) to catalyze the transesterification of palm oil with methanol for biodiesel production. TiO_2_ of nanometer particle size was used to increase the contact area between the catalyst and the reactants. The TiO_2_/CH_3_ONa photocatalyst was irradiated by UV light from light-emitting diode (LED) lamps with a wavelength range of 320–380 nm to improve methyl ester production. The parameters such as the alcohol/oil molar ratio, amount of catalyst added, with and without UV irradiation, reaction temperature, and reaction time were varied to produce the palm oil biodiesel. The fuel properties of the biodiesel produced from various production conditions were analyzed to find the optimum reaction conditions.

## 2 Experimental Details

Palm oil was reacted with methanol in various molar ratios under the assistance of the TiO_2_/CH_3_ONa photocatalyst irradiated with ultraviolet light from LED lamps to proceed with the transesterification for biodiesel production. The experimental details including the materials, preparation method for biodiesel, analyzing equipment, and procedures are expressed in the following.

### 2.1 Preparation Method of the Photocatalyst for Biodiesel Production

Sodium methoxide (CH_3_ONa) has been widely used as a strong alkaline catalyst to enhance transesterification reactions for industrial biodiesel production. CH_3_ONa of 5 M concentration was provided by Merck Taiwan Ltd. (Taipei City, Taiwan). TiO_2_ powder is full of porosity structure, which can be used to embed the alkaline catalyst to have combined catalyst functions. The commercial TiO_2_ nanoparticles were vended by Uni-Onward Corp., Ltd. (New Taipei City, Taiwan).

TiO_2_ of the anatase phase with 99.5% purity, which is an n-type semiconductor, bears excellent photocatalytic activity for chemical reactions. The average particle size was about 22 nm, and the specific surface area (i.e., Brunauer–Emmett–Teller (BET) surface area) of TiO_2_ nanoparticles can be as large as 50.5 m^2^/g. One gram of nanoparticles of TiO_2_ with a concentration of 0.1 M were immersed directly into 10 mL of an aqueous CH_3_ONa solution for 10 h. The photocatalyst TiO_2_ impregnated with the strong alkaline catalyst CH_3_ONa was prepared. Aqueous CH_3_ONa solution was mixed with TiO_2_ powder of 0.1 M concentration so that the CH_3_ONa compound flowed and filled in the pores and holes of TiO_2_. The impurities in the aqueous mixture of TiO_2_ and CH_3_ONa were removed by flowing the solution through a funnel and a glass fiber.

The mixture was then filtered through a ceramic funnel and a glass fiber by a vacuum pump to remove the impurities. The wet photocatalyst of TiO_2_ embedded with CH_3_ONa was heated at 100 °C to distill away volatile compounds and water and calcined at six various temperatures from 150 to 450 °C in a furnace (Model DF404, Deng Yng Ltd., New Taipei City, Taiwan) to calcine the combined catalyst preparation. The calcined catalyst was first heated to 100 °C in 0.5 h. This temperature was maintained for 4 h. The temperature was then increased to 200 °C in 2 h and then maintained for 3 h to finally complete the preparation of the calcined catalyst.

### 2.2 Biodiesel Production Assisted with the Irradiation of Ultraviolet Light on the Photocatalyst

In this experiment, ultraviolet (UV) light from LED lamps with wavelengths between 320 and 380 nm was used to irradiate the solid alkaline catalyst TiO_2_/CH_3_ONa during the transesterification reaction from palm oil to biodiesel. The irradiation power of the ultraviolet (UV) light from LED lamps was set at 200 mW. The effects of UV-light irradiation onto the TiO_2_/CH_3_ONa photocatalyst on the conversion ratio of the palm oil biodiesel and their fuel properties were evaluated to find the optimum reaction conditions including UV-light irradiation, reaction time and temperature, molar ratio of methanol/palm oil, etc. The experimental procedures of the transesterification reaction irradiated with UV light from LED lamps are described as above.

When the transesterification was completed, an adjusted amount of glacial acetic acid was added to the crude biodiesel mixture to neutralize the product. A centrifuging machine was then used to separate the crude product into biodiesel and glycerol at the top and bottom layers, owing to their significant density difference. The separated biodiesel was then water-washed by deionized water three times, followed with heating at 110 °C to remove water, methanol, and other volatiles. The obtained biodiesel was thereafter used as the fuel sample in this study.

### 2.3 Biodiesel Properties Produced through Ultraviolet-Light Irradiation on the Photocatalyst

An oxygen bomb calorimeter (Model 1261, Parr Inc., Moline, IL) was used to analyze the heating values of various fuel samples in units of MJ/kg. The definition of the heating value is the quantity of heat released from the complete burning of a tested fuel. The kinematic viscosity of the fuel sample was measured by a viscometer (Model K698, Cannon Instrument Ltd., State College, PA), which was buried in a water bath filled with liquid fuel at a constant temperature of 40 °C. The required time (*t*) to flow between indicated marks was noted. The multiplication of the flowing time (*t*) and coefficient *k*, which is 0.001045 mm^2^/s^2^ for the type of U-tube viscometer, are the required parameters to calculate the kinematic viscosity.

A volumetric Karl Fischer-type moisture titrator (Model DL-31, Mettler-Toledo Inc., Greifensee Schweiz, Switzerland) was used to analyze the moisture content in the feedstock oil or biodiesel product based on the Coulomb electricity method with ISO 12973. Excess water content in liquid fuel might reduce the heat release and corrode the engine parts, leading to deterioration of the engine performance.^30^

The acid value of a lipid indicates the extent of rancidity due to the existence of free fatty acids. The acid value of the fuel sample was analyzed by an automatic titrator (Model 785 DMP Titrino, Metrohm AG, Herisau, Switzerland) based on the amount of titrant required to neutralize the fuel sample.

A distillation temperature analyzer (Model HAD-620, Walter Herzog GmbH, Lauda-Königshofen, Germany) was used to acquire the distillation temperature distribution of the fuel sample. Fossil fuel or biodiesel consists of many different hydrocarbon compounds that form various boiling points. The first drop, 10, 20, 50, 90 vol %, and the last drop are heated, distilled, and condensed at the corresponding temperatures marked as *T*_IBP_, *T*_10_, *T*_20_, *T*_50_, *T*_90_, and *T*_EP_, respectively. *T*_IBP_ and *T*_EP_ are abbreviations of the initial boiling point and end point, respectively. The specific gravity (sg) of the biodiesel sample was measured by a liquid-phase hydrometer stored in a tank containing 100 mL of liquid fuel sample. The API gravity (*G*) can be converted from sg by the following equation
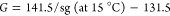
1

The cetane index, which is an alternative to the cetane number to indicate the difficulty extent of compression ignition for a fuel used in diesel engines, can be calculated based on the equation below
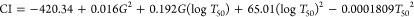
2

A gas chromatograph (Model GC-14a, Shimadzu Ltd., Chiyoda-ku, Tokyo, Japan) and a Flame Ionization Detector (FID) worked together to analyze the content of fatty acid methyl esters. The length, inside diameter, and film thickness of the separation columns are 0.25 μm, 0.32 mm, and 30 m in the dimensions of film thickness, inside diameter, and length. The carrier gas used for GC analysis was nitrogen. The software of the chromatography data system (CDS) was applied to calculate the content of FAME. The mean values were calculated by repeating each experiment 3–5 times. The experimental uncertainties of the FAME content, water content, acid value, kinematic viscosity, distillation temperature, and heating value were 3.61, 2.95, 1.57, 2.63, 1.91, and 2.75%, respectively.

## 3 Results and Discussion

The biodiesel was prepared through a transesterification reaction of palm oil with methanol under various molar ratios assisted with catalytic conversion of a photocatalyst TiO_2_ synthesized with a strong alkaline catalyst CH_3_ONa. The ultraviolet (UV) light of LED (i.e., light-emitting diode) lamps was used to irradiate the photocatalyst to induce its jump from the valence energy band to conduction energy band so that the photocatalytic effects can be excited to facilitate the chemical reaction. The effects of the irradiation of UV light upon the catalyst surface on the fuel properties of the biodiesel prepared through various reaction conditions were analyzed and discussed below. Photocatalysts that a catalyst carrier impregnated with a strong alkaline or acid catalyst to have combined functions of various catalysts might have various compositions and catalyst characteristics. The FAME yields from various feedstock oils and photocatalysts are compared in Table 1(31−39) The FAME yields lie in the range between 92 and 98%. Edible oil such as palm oil, inedible oil like Jatrophas oil, and new-generation feedstock oil such as microalgal lipids could be used to produce renewable biodiesel fuel.

**Table 1:** Comparison of Biodiesel Yield by Heterogeneous Photocatalysts^31−39^

feedstock	photocatalyst	synthesis method	biodiesel yield (%)	reusability (cycles)	refs
canola oil	Li/TiO_2_	wet impregnation	98	4	(44)
soybean oil	Li_2_TiO_3_	solid state	98.55	4	(45)
waste cooking oil	TiO_2_–MgO	sol–gel	92.3	4	(46)
linseed oil	TiO_2_/C_4_H_4_KO_6_	impregnation	98.54	5	(47)
waste cooking oil	MgMoO_4_/TiO_2_	impregnation	97.4	>10	(48)
soybean oil	Sr–Ti mixed oxide	sol–gel	98	4	(49)
palm oil	SrTiO_3_	sol–gel	93.14	5	(50)
dairy scum oil	Ca–Ti nanocatalyst	hydrothermal	97.2	5	(51)

### 3.1 Effects of TiO_2_/CH_3_ONa Catalysts Irradiated with UV Light on the Formation of Fatty Acid Methyl Esters

**Figure 1:**
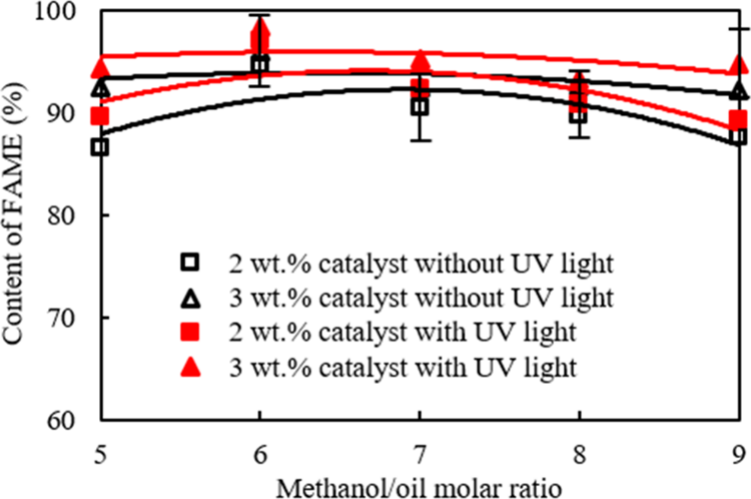
Effects of different alcohol/oil molar ratios and catalyst amounts on methyl ester content under illumination.

Figure 1 shows the effects of different methanol/palm oil molar ratios and catalyst amounts on the fatty acid methyl ester (FAME) content under ultraviolet (UV) light irradiation on the TiO_2_/CH_3_ONa catalyst surface. It can be seen that the FAME formation through the transesterification reaction reached 98.4% under 3 wt % catalyst addition and alcohol/oil molar ratio equal to 6.

The strong alkaline catalyst CH_3_ONa has been considered to be a promising catalyst to enhance the biodiesel yield. Chen et al.^40^ found that the biodiesel yield with a neat CH_3_ONa catalyst is higher than that with a NaOH catalyst. Lin and Lin^41^ prepared soybean oil biodiesel through a transesterification reaction catalyzed by a 1 wt % strong alkaline catalyst CH_3_ONa by weight of soybean oil at a reaction temperature of 60 °C for 1 h to complete the process. The yield of fatty acid methyl esters can reach 98.7 wt %. In contrast, neat TiO_2_ particles play no effect on catalyzing biodiesel production. However, the adequate combination of TiO_2_ nanoparticles (NPs) with other metallic or nonmetallic materials is very efficient and selective for biodiesel production.^42^

The lowest FAME content, which was 86.5 wt %, appeared when the molar ratio and catalyst addition were 1 and 2 wt %, respectively, without UV-light irradiation. In contrast, after the catalyst amount was increased to 3 wt % and the molar ratio increased to 6 and under UV-light irradiation, there was the highest FAME formation, which reached 98.4 wt %. UV-light irradiation significantly facilitated the formation of fatty acid methyl esters. The FAME contents were increased from 95.95 wt % under the reaction conditions of the molar ratio and catalyst amount of 6 and 3 wt % without UV-light irradiation to 98.4 wt % under the same molar ratio and catalyst amount but with UV-light irradiation. This implied that the FAME formation considerably increased by 2.55% under irradiation with UV light.

The exchange process of organic alkyl groups (R_1_, R_2_, and R_3_) of vegetable oil with methyl alcohol occurs during the transesterification.^43^ The strong alkaline catalyst CH_3_ONa provides the alkoxide to facilitate the nucleophilic attack on the carbonyl carbon of vegetable oil to produce a tetrahedral intermediate, which proceeds to the formation of FAME and glycerol. The existence of titanium dioxide would induce a photocatalytic reaction to transfer electrons from the valence energy band to the conduction energy band, leading to the enhancement of the transesterification reaction and thus biodiesel production.

The TiO_2_/CH_3_ONa catalyst enhanced the continuous dissociation of methanol to release OH^–^ and H^+^ radicals during the transesterification reaction.^44^ The catalyst CH_3_ONa carried by TiO_2_ powder was prone to capture OH^–^ radicals under ultraviolet (UV) light irradiation to incur the photocatalytic reaction.^45^ The TiO_2_/CH_3_ONa catalyst after absorbing energy from the irradiation of UV light, the electrons deliver energy from the valence energy band to the conduction energy band. The electrons then return to the valence energy band to associate with the electron holes and in turn release light and heat to return to the low-energy state. The electrons then capture OH^–^ radicals to repeat the photocatalytic reaction. During the process, additional energy was continuously released to enhance the transesterification conversion efficiency significantly.

The compositions of fatty acid methyl esters produced through the transesterification reaction under the molar ratio of 6 and catalyst amounts of 2 and 3 wt % under UV-light irradiation are shown in Table 2. The saturated fatty acids and the combined fatty acids ranging from C14 to C21 were found to significantly increase with the increase of the catalyst amounts from 2 to 3 wt %. Diesel fuel is generally composed of hydrocarbon compounds and most carbon chains are in the range between C16 and C18.^46^ The hydrocarbon compounds containing carbon chains of C16–C18 in the fatty acid methyl esters of the palm oil biodiesel produced under the molar ratio of methanol/palm oil equal to 6 and the catalyst amounts of 2 and 3 wt % were 93.66 and 94.91 wt %, respectively. It is inferred that the biodiesel made from the latter condition is more adequate to be an alternative fuel to diesel fuel. However, both palm oil biodiesels from those two manufacturing conditions can be promising liquid fuels for diesel engines.

**Table 2:** Weight Percentages (wt %) of Biodiesel Made from a Molar Ratio of Methanol/Palm Oil of 6 Added with the Catalyst TiO_2_/CH_3_ONa of 2 and 3 wt % of the Weight of Palm Oil under the Irradiation of Ultraviolet Light

	catalyst addition (wt %)	
composition of fatty acid methyl ester	2	3
C14–C24	97.06	98.4
C12:0	3.26	3.10
C14:1	0.09	0.12
C16:0	34.96	35.07
C16:1	0.56	0.45
C18:0	4.33	5.29
C18:1	37.13	36.35
C18:2	16.12	17.34
C18:3	0.56	0.41
C20:0	0.03	0.20
C21:0	0.02	0.16
saturated fatty acids	42.60	43.82
unsaturated fatty acids	54.46	54.67

**Figure 2:**
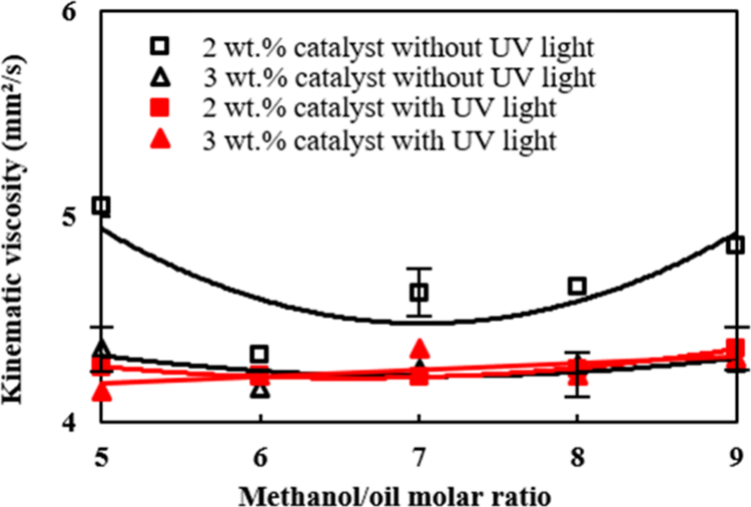
Effects of different methanol/palm oil molar ratios and catalyst amounts on the kinematic viscosity under irradiation of ultraviolet light.

Kumar et al.^47^ prepared biodiesel from waste cooking oil (WCO) catalyzed by a glycerol-derived sulfonated carbon catalyst (SCG). They found that the SCG catalyst maintained the highest fatty acid methyl ester (FAME) yield higher than 90% after two consecutive cycles for reusability. Kim et al.^48^ used a combined catalyst of CH_3_ONa with a gel-type resin Monosphere catalyst to transesterify soybean oil with methanol. They found that the FAME yield of the regenerated catalyst lost 5% compared to the fresh one. Our reusability results for the catalyst also indicated 5% reduction of FAME yield for each used catalyst. Each fresh catalyst might be used in transesterification for four consecutive cycles to retain the FAME yield greater than the acceptable 80% (Figure 2).

### 3.2 Effects of TiO_2_/CH_3_ONa Catalysts Irradiated with UV Light on the Kinematic Viscosity

**Figure 3:**
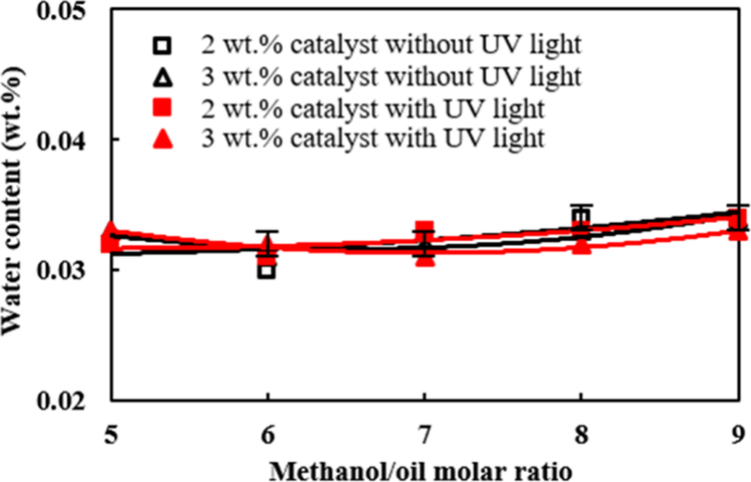
Effects of different alcohol/oil molar ratios and catalyst amounts on the water content under irradiation with ultraviolet light.

Figure 3 shows the effects of different methanol/palm oil molar ratios and catalyst amounts on the kinematic viscosity under UV-light irradiation on the reactant mixture. The kinematic viscosities of the produced biodiesel were within the range of 4–6 mm^2^/s, which lie within the biodiesel specification of kinematic viscosity of EN 14214.^49^Table 2 shows the fatty acid methyl ester compositions of the palm oil biodiesel produced under the molar ratio of 6 and the catalyst amounts of 2 and 3 wt % irradiated with UV light. Both the total methyl ester and the saturated fatty acid contents increased significantly with the increase of the catalyst addition from 2 to 3 wt % in Table 2. The increase of the catalyst amount from 2 to 3 wt % under UV-light irradiation rendered a greater extent of complete transesterification. UV-light irradiation on the TiO_2_/CH_3_ONa catalyst surface catalyzes the transesterification reaction, leading to considerably larger FAME formation than that without UV-light irradiation, which are 98.4 and 95.95 wt %, respectively, as seen in Table 2. This implies that more triglycerides in the raw palm oil were converted to fatty acid methyl esters under UV-light irradiation^50^ on the catalyst surface during transesterification. The acid value in turn was reduced, and the cetane index increased significantly under the case of UV-light irradiation in Table 3.

**Table 3:** Comparison of Biodiesel Properties Prepared with and without Ultraviolet-Light Irradiation on the 3 wt % Solid Alkali Catalyst and a Methanol/Palm Oil Molar Ratio of 6

	catalyst addition (wt %)	
fuel property	light illumination	no light illumination
content (wt %) of fatty acid methyl ester	98.4	95.95
acid value (mg KOH/g)	0.21	0.32
kinematic viscosity (mm^2^/s)	4.23	4.17
heating value (MJ/kg)	39.75	40.02
specific gravity of API	31.89	30.77
highest distillation temperature	355	354
cetane index	51.01	50.05

The irradiation of UV light also facilitated the decomposition of water in the reactant mixture into OH^–^ and H^+^ radicals^51^ due to the photocatalytic effect. The unsaturated fatty acids in the palm oil biodiesel then reacted with the H^+^ radicals to form saturated fatty acids, leading to higher kinematic viscosity in comparison with that from the TiO_2_/CH_3_ONa catalyst without UV-light irradiation, as seen in Figure 2.

### 3.3 Effects of TiO_2_/CH_3_ONa Catalysts Irradiated with UV Light on the Moisture Content and Acid Value

**Figure 4:**
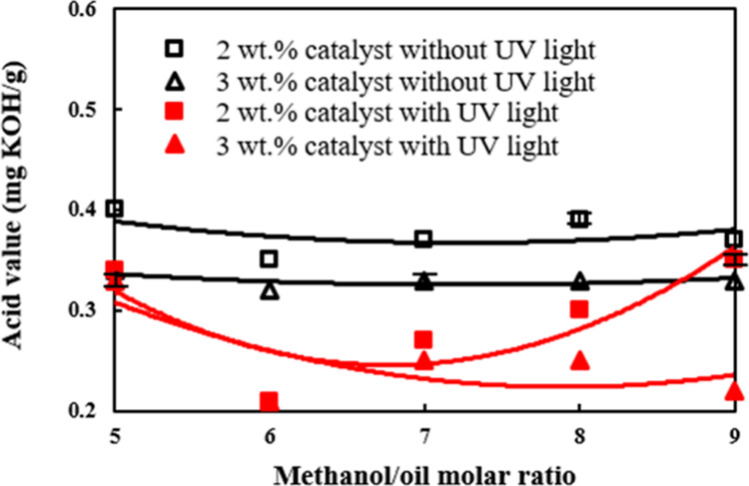
Effects of different methanol/palm oil molar ratios and catalyst amounts on the acid value under the irradiation of ultraviolet light.

Figure 4 shows the effects of different methanol/palm oil molar ratios and catalyst amounts on the water content in the palm oil biodiesel under UV-light irradiation. The water content of the biodiesel prepared through transesterification reaction under various molar ratios, catalyst amounts, and with and without UV-light irradiation was less than 0.04 wt %, and there was no significant difference among those preparing conditions. However, the palm oil biodiesel prepared at a molar ratio of 6 was shown to have the least water content among those molar ratios from 5 to 9. In addition, the increase of the catalyst from 2 to 3 wt % together with UV-light irradiation was found to have a slightly lower water content. This is primarily owing to a larger complete extent of transesterification^52^ when the molar ratio and catalyst addition were equal to 6 and 3 wt %, respectively, associated with UV-light irradiation on the reactant mixture.

The effects of molar ratio, catalyst amount, and UV-light irradiation on the acid value of the palm oil biodiesel are shown in Figure 4. The production rate of methyl esters was the highest, which was 98.4 wt % when 3 wt % of the TiO_2_/CH_3_ONa photocatalyst was added into the reactant mixture that was irradiated with UV light in Figure 1. In contrast, the lowest acid value, which was 0.21 KOH mg/g biodiesel, occurred when 2 wt % of the photocatalyst by weight of palm oil was added to the reactant mixture, and the molar ratio of methanol/palm oil of 6 was used as shown in Figure 4. The preparing condition as stated above, which was shown to have the lowest acid value in Figure 4, is exactly the same to have the lowest water content in the palm oil biodiesel in Figure 3. Hence, it is inferred that the lower acid value is associated with the lower water content in the biodiesel^53^ and these two properties have similar variation trends with the molar ratio of alcohol/feedstock oil. In contrast, the trend of the fatty acid methyl ester (FAME) formation with the molar ratio of methanol/feedstock oil is adverse to those of the water content and acid value.

### 3.4 Effects of TiO_2_/CH_3_ONa Catalysts Irradiated with UV Light on the Distillation Temperature and Cetane Index

**Figure 5:**
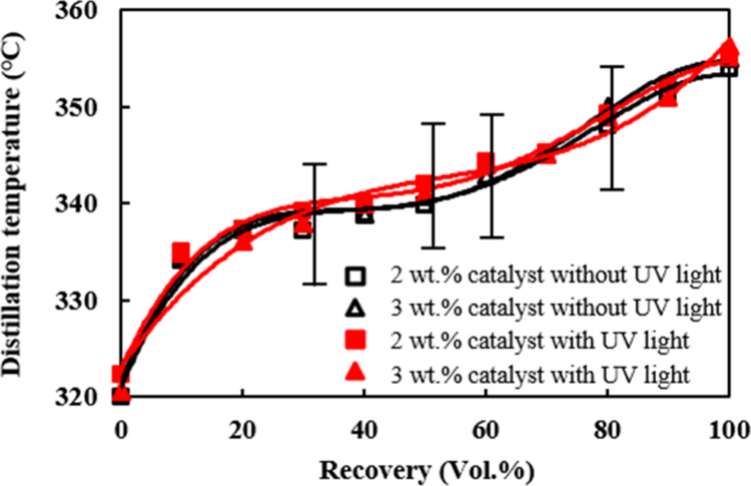
Distillation temperature curve for the biodiesel made under a methanol/palm oil molar ratio of 6 and catalyst amount of 3 wt % under ultraviolet-light illumination.

**Figure 6:**
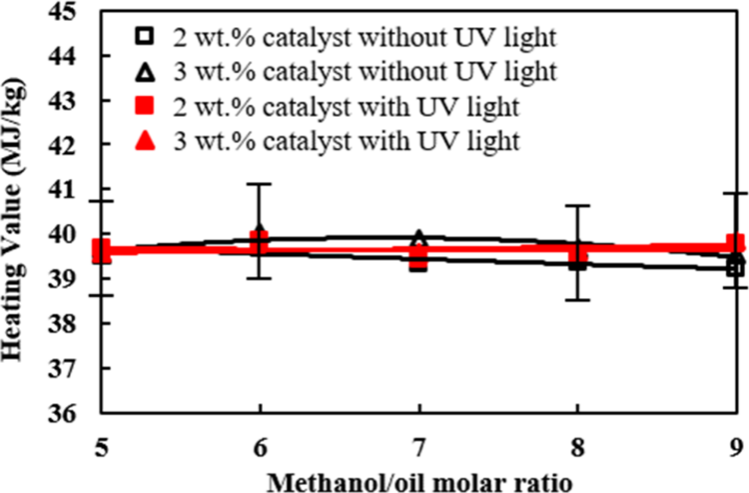
Effects of different methanol/palm oil molar ratios and catalyst amounts on the heating value of biodiesel made under ultraviolet-light illumination.

The variations of the distillation temperature curves with the amount of the catalyst addition and UV-light irradiation are shown in Figure 5. The molar ratio of methanol/palm oil was set at 6. The distillation temperatures of biodiesel were found to increase with the increase of the catalyst addition from 2 to 3 wt % and the irradiation of UV light in Figure 6. The contents of saturated fatty acids were increased from 42.60 to 43.82 wt %, and meanwhile, the hydrocarbon compounds with longer carbon chains from C16 to C18 increased from 93.66 to 94.91% with the catalyst addition from 2 to 3 wt %, as revealed in Table 2. The distillation temperature thus increased under the preparing conditions of 3 wt % of the catalyst addition and irradiation of UV light to facilitate larger formations of both saturated fatty acids and compounds with longer carbon chains.^54^

The cetane indices of the biodiesel prepared with 3 wt % catalyst, molar ratio of methanol/palm oil equal to 6, and with or without UV-light irradiation were compared in Table 2. The cetane index of the biodiesel prepared with UV-light irradiation was 51.01, which was higher than that without the light irradiation, which was 50.05. Tamilselvan et al.^55^ proposed that the cetane index was influenced by the carbon-chain structure and composition of saturated fatty acids. The increase in the content of long carbon-chain fatty acids leads to an increase in the cetane index of the biodiesel.

Both the contents of saturated fatty acids and fatty acids with the carbon chain in the range between C16 and C18 increased when the catalyst addition increased from 2 to 3 wt %. In consequence, the cetane index increased accordingly in Table 3.

### 3.5 Effects of TiO_2_/CH_3_ONa Catalysts Irradiated with UV Light on the Heating Value

The heating values of the palm oil biodiesel prepared under various catalyst amounts and molar ratios of methanol/palm oil and with or without UV-light irradiation were compared in Figure 6. The heating values of the biodiesel samples varied slightly in the range between 39 and 40 MJ/kg under various preparing conditions. A higher heating value of 39.85 MJ/kg was observed when the molar ratio of methanol/palm oil and catalyst amount were equal to 6 and 3 wt %, respectively. The heating value could be increased by 150 kcal/mol after a feedstock oil is converted to its biodiesel product through the transesterification reaction.^56^ The irradiation of UV light on the photocatalyst TiO_2_/CH_3_ONa incurred a higher extent of transesterification reaction, leading to an increased formation of fatty acid methyl esters as shown in Figure 1, and a higher heating value in Figure 6 accordingly. However, UV-light irradiation on the photocatalyst TiO_2_/CH_3_ONa did not alter the elemental compositions of carbon and hydrogen of the biodiesel. Hence, the heating value of the biodiesel was not significantly changed in Figure 6.^57^

## 4 Conclusions

In this experiment, the solid alkaline catalyst TiO_2_/CH_3_ONa was used to catalyze the conversion of palm oil into biodiesel under different catalyst amounts and methanol/palm oil molar ratios to find the optimal experimental conditions. The catalyst was irradiated with ultraviolet (UV) light from light-emitting diode (LED) lamps with wavelengths ranging from 320 to 380 nm to enhance the transesterification reaction. The characteristics of the fatty acid methyl esters formed from the irradiation of UV light were compared with those without UV-light irradiation. The main experimental results are summarized as follows.

1. Under ultraviolet (UV) irradiation on the reactant mixture, the conversion rate from the raw palm oil to the biodiesel reached the highest, which was 98.4 wt % when the reaction was carried out at 3 wt % of the strong alkaline catalyst addition and 6 of the molar ratio of methanol/palm oil. The lowest formation of fatty acid methyl esters occurred at a molar ratio of 5. The content of fatty acid methyl esters slightly decreased to 97.06 wt % when 2 wt % catalyst, molar ratio of 6, and UV-light irradiation were used.
2. The kinematic viscosities of the biodiesel prepared by adding 2 and 3 wt % of the catalyst by weight of palm oil under UV-light irradiation were in the range between 4 and 5 mm^2^/s, which are within the limits of the kinematic viscosity specification of the EN 14214 standard. The lowest kinematic viscosities of 4.23 mm^2^/s were found at the reaction of methanol/oil molar ratio of 6 and 3 wt % of TiO_2_/CH_3_ONa catalyst irradiated with ultraviolet light.
3. The irradiation of ultraviolet (UV) light caused no obvious difference in the water contents in the palm oil biodiesel prepared with or without UV-light irradiation when 2–3 wt % catalyst of the palm oil weight was added. This might be ascribed to the fact that the distillation and water removal processes during the biodiesel production significantly reduced the water content in the biodiesel product.
4. The acid value of the palm oil biodiesel prepared by ultraviolet-light irradiation on the 3 wt % TiO_2_/CH_3_ONa catalyst of the palm oil weight and molar ratio of methanol/palm oil equal to 6 was shown to be the lowest, which was 0.21 KOH mg/g biodiesel and significantly lower than those biodiesels made from the same amount of catalyst addition and molar ratio of methanol to palm oil of 6 but without UV-light irradiation.
5. The irradiation of ultraviolet light on the reactants added with 3 wt % strong alkaline TiO_2_/CH_3_ONa catalyst under the molar ratio of methanol/palm oil equal to 6 during the transesterification resulted in the highest distillation temperature, which reached 356 °C in comparison with other biodiesels prepared without ultraviolet-light irradiation. There were slightly higher distillation temperature distributions for the biodiesel made from the reactant mixture with ultraviolet-light irradiation than those made without UV-light irradiation. Moreover, the cetane index for the biodiesel with the light irradiation prepared with 3 wt % catalyst and 6 of the molar ratio appeared to be the highest, 51.01, among the biodiesel made with and without UV-light irradiation and various amounts of the catalyst addition.
6. The heating values of the biodiesel samples prepared by adding 2 and 3 wt % of the catalyst irradiated by UV light and the molar ratio of methanol/palm oil equal to 6 were found to be higher, which were 39.85 and 39.75 MJ/kg, respectively, than those biodiesels prepared without UV-light irradiation. The heating values of all of the biodiesel samples prepared by the catalyst additions from 2 to 3 wt % and molar ratios of methanol to raw oil from 5 to 9 were shown to be lower than those biodiesels made under the molar ratio of alcohol to oil of 6.

## Nomenclature

CaO: calcium oxide
CDS: chromatography data system
CH_3_ONa: sodium methoxide
FID: flame ionization detector
KOH: potassium hydroxide
MTBE: methyl tertiary-butyl ether
NaOH: sodium hydroxide
SCM: supercritical methanol
THF: tetrahydrofuran
TiO_2_: titanium dioxide
UV: ultraviolet
